# Efficacy of naproxen prophylaxis for the prevention of heterotopic ossification after hip surgery: a meta-analysis

**DOI:** 10.1186/s13018-018-0747-8

**Published:** 2018-03-05

**Authors:** Ran Ma, Guan-Hong Chen, Liu-Jing Zhao, Xi-Cheng Zhai

**Affiliations:** Department of Orthopedics, Shanxian Central Hospital, No.1 Wenhua Road, Shanxian, Shandong 274300 China

**Keywords:** Naproxen, Hip surgery, Heterotopic ossification, Meta-analysis

## Abstract

**Background:**

This meta-analysis aimed to assess whether the specific nonsteroidal anti-inflammatory drug (NSAID) naproxen has a role in reducing the occurrence of heterotopic ossification after hip surgery.

**Methods:**

Potential studies were identified in the following electronic databases: PubMed, EMBASE, Web of Science, Cochrane Library, and Google. We included studies involving hip surgery patients in which the intervention group received naproxen and the control group received placebo. The occurrence of heterotopic ossification and complications were the final outcomes. Stata 13.0 was used for the meta-analysis.

**Results:**

Four randomized controlled trials (RCTs) involving 269 patients were ultimately included in this meta-analysis. The use of naproxen was associated with a significant reduction in the occurrence of heterotopic ossification at 1.5-, 3-, 6-, and 12-month follow-ups (*P* < 0.05). There was no significant difference in the occurrence of complications between treatment and control groups (*P* > 0.05).

**Conclusion:**

Our analysis indicates that naproxen can decrease the occurrence of heterotopic ossification without increasing complications in hip surgery patients. Due to the limited number of studies included, more high-quality RCTs are needed to identify the optimal dose of naproxen.

## Background

Heterotopic ossification (HO) was one of the most common complications of hip surgery. Indeed, HO, which is mainly induced by an inflammatory response to surgery, undermines the intended benefits of surgery [1, 2]. Due to variability in hip surgical techniques, the incidence of HO after such a procedure varies from 0 to 73% [3, 4]. Histologically, HO consists of lamellar bone derived from abnormity activity of osteoblast cells in atypical locations [5]. HO decreases range of motion (ROM) and causes hip pain, swelling, and joint stiffness.

Nonsteroidal anti-inflammatory drugs (NSAIDs) and radiotherapy are often used to reduce the occurrence of HO after hip surgery [6, 7]. Although NSAIDs have been widely used for HO prophylaxis, the risk of gastrointestinal side effects has drawn the attention of surgeons. Thus, radiotherapy may be a preferred option in very high-risk patients or in those with contraindications to NSAIDs. Nonetheless, NSAIDs are considerably more cost effective than radiotherapy.

Different drugs have been used for HO prophylaxis, though NSAIDs have been the mainstay [7], with greatly variable efficacy. The efficacy of naproxen, a type of NSAID, for the prophylaxis of HO after hip surgery remains in debate, and there is yet no relevant meta-analysis on the use of naproxen in this application.

The objective of this meta-analysis was to evaluate the efficacy of naproxen for prophylaxis of HO after hip surgery.

## Methods

### Literature search

Both published and unpublished literature was identified in the following databases: PubMed (1950–November 2017), EMBASE (1974–November 2017), Cochrane Library (November 2017 Issue 3), and Google Scholar (1950–November 2017). The MeSH terms and combinations of terms used in the search were as follows: “THA” OR “THR” OR “total hip arthroplasty” OR “total hip replacement” OR “Arthroplasty, Replacement, Hip”[Mesh] OR “hip arthroscopy” AND “naproxen” [Mesh terms] OR “Naproxen”[Mesh]. Reference lists of included studies were scrutinized for other relevant publications. Only articles originally written in English or translated into English were considered. When multiple reports describing the same sample were published, the most recent or complete report was utilized. As this meta-analysis collected data from published articles, no ethics approval was necessary for the research.

### Inclusion and exclusion criteria

Preferred Reporting Items for Systematic Reviews and Meta-analyses (PRISMA) guidelines were followed for the inclusion of studies in this systematic review and associated meta-analysis [8]. The studies had to meet the following standards to qualify: (1) a randomized controlled trial (RCT) design; (2) intervention using naproxen for prophylaxis of HO; (3) a control group receiving either placebo or no intervention; (4) reporting of HO outcomes, including incidence of HO at 1.5, 3, 6, or 12 months after surgery, as well as potential complications. The following exclusion criteria were applied: (1) animal experiments or case reports, (2) failure to provide the final results of interest (incidence of HO at 1.5, 3, 6, or 12 months after surgery, as well as potential complications), and (3) repeated or overlapping publications.

### Data collection and outcome measures

Two researchers independently extracted the following data from each study that met inclusion criteria: first author, year of publication, country, participant demographic characteristics, and treatment regime for each group. Discrepancies were resolved by consensus. The primary outcome measure for this meta-analysis was the appearance of radiographically determined HO during follow-up. Secondary measures were side effects related to the study medication.

### Quality assessment

The methodological quality of the included trials was assessed by Cochrane Collaboration’s tool [9, 10]. The following items were assessed: random sequence generation, allocation concealment, blinding of participants, personnel and outcome assessment, incomplete outcome measures, selective outcome reporting, and other bias. Two independent practitioners independently screened and reviewed every entry for accuracy and consistency, and any discrepancies were resolved by consensus.

### Statistical analysis

Stata software, version 13.0 (Stata Corp., College Station, TX), was used to perform the meta-analysis. For dichotomous variables, risk ratios (RRs) with the corresponding 95% confidence intervals (CIs) were calculated; weighted mean differences (WMDs) were used for numerical variables. Where significant heterogeneity was found, data were pooled using a random-effect model. Statistical heterogeneity among individual studies was evaluated based on Cochrane’s *Q* test and the *I*^2^ index, and statistical heterogeneity was confirmed if *I*^2^ was above 75% and *P* < 0.10 [11]. Publication bias was evaluated using the Egger regression asymmetry test [12]. Results were considered statistically significant when the *P* value was less than 0.05.

## Results

### Trial characteristics

**Fig. 1:**
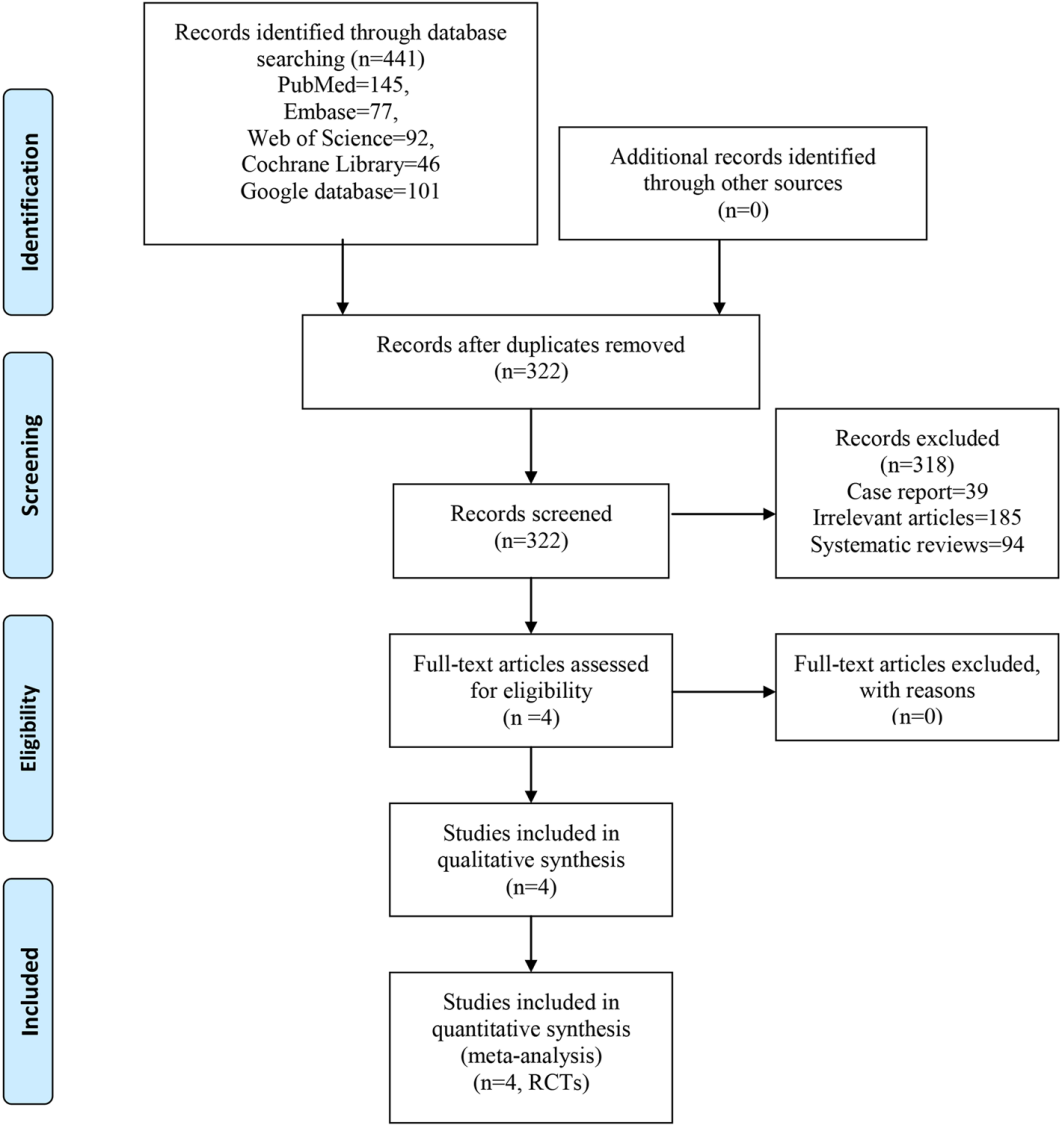
PRISMA flowchart for the included studies

We retrieved 64 relevant reports from electronic databases; of these, we identified 13 eligible studies for further assessment. Four RCTs [13–16] involving 269 patients finally met the predetermined inclusion criteria, as illustrated in Fig. 1. All studies reported statistically significant differences in the incidence of HO between patients treated with naproxen and control subjects. Patient demographic details were balanced between medication and control groups in the four included studies. The sample size among trials ranged from 50 to 108. The method of administration of medication varied in dosage and course among the trials. Detailed characteristics of the relevant literature are presented in Table 1.

**Table 1:** The general characteristic of the included studies

Author	Country	Sample (I/C)	Age (I/C)	Surgery	Intervention	Control	Outcomes	Follow-up (weeks)	Study
Beckmann 2015	USA	54/54	35.1/35.1	Hip arthroscopy	Naproxen (500 mg, twice daily, total 3 weeks	Placebo	1,2,3,4,5	3	RCTs
Vielpeau 1999	France	28/28	66/62.8	THA	Naproxen (250 mg, 3 times daily, total 6 weeks)	Placebo	1,2,3,5	6	RCTs
Gebuhr 1991	Denmark	28/27	75/70	THA	Naproxen (500 mg twice on operation day, 250 mg, 3 times daily, total 4 weeks)	Placebo	2,3,5	4	RCTs
Gebuhr 1995	Denmark	27/23	72/73	THA	Naproxen (500 mg twice daily for 7 days from operation day on)	Placebo	1,2,3,4	12	RCTs

1: the occurrence of HO at 1.5 months after surgery; 2: the occurrence of HO at 3 months after surgery; 3: the occurrence of HO at 6 months after surgery; 4: the occurrence of HO at 12 months after surgery; 5: the occurrence of complications

*I* intervention group, *C* control group, *THA* total hip arthroplasty

**Fig. 2:**
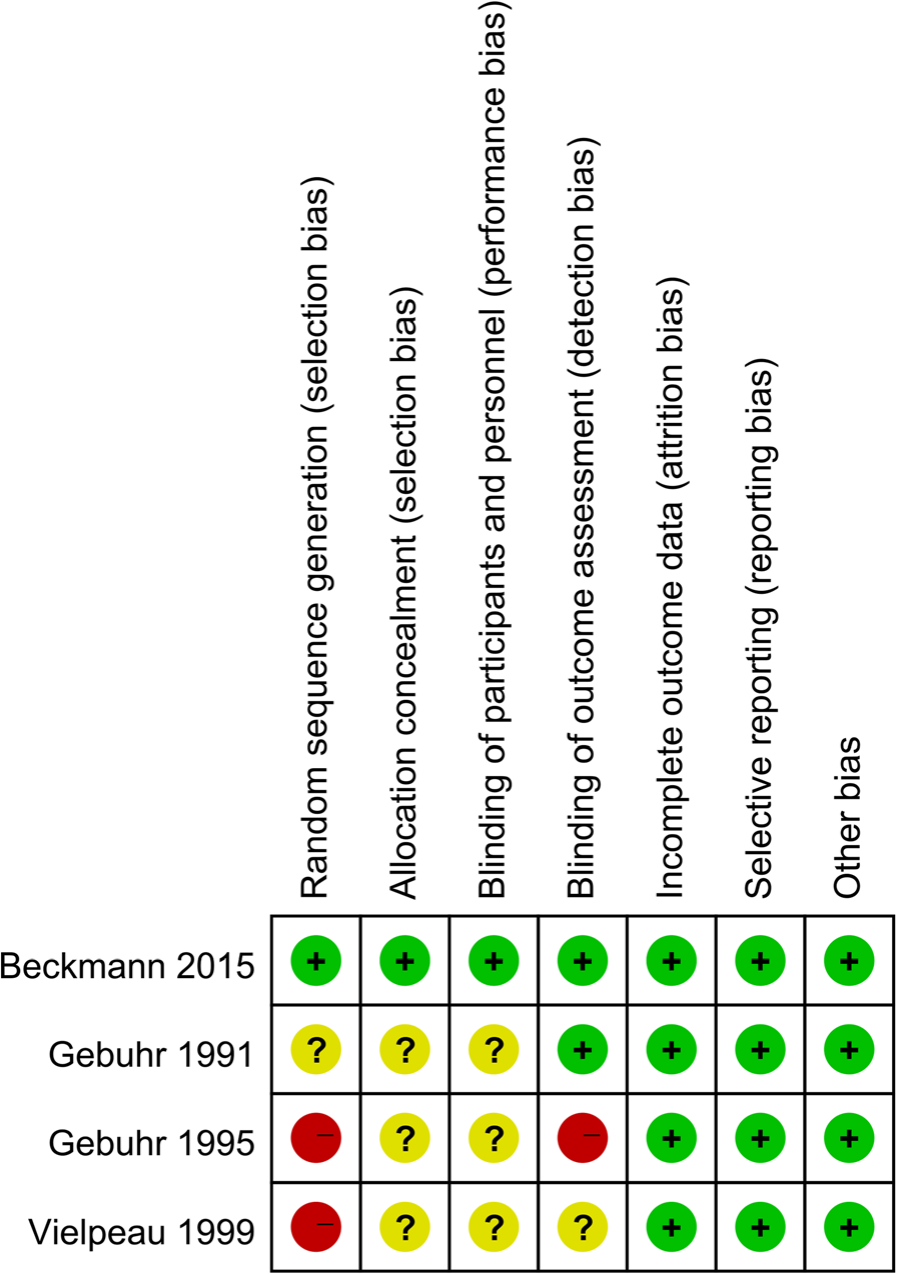
Risk of bias summary for the included studies

**Fig. 3:**
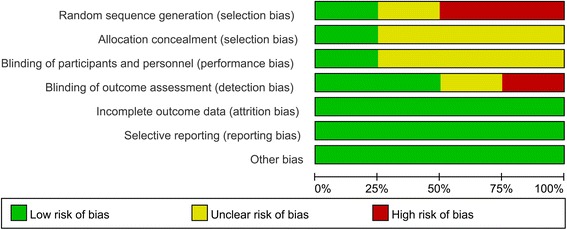
Risk of bias graph for the included studies

The methodological quality of the included trials is summarized in Figs. 2 and 3. All included studies were described as RCTs. Only one study reported acceptable methods of randomization and clearly described the method of allocation concealment [13]. Three studies [14–16] reported blinding of participants and personnel, whereas one of the trials conducted by Gebuhr [16] provided no details in this regard. Selective outcome reporting bias was present, as enrollment of participants was consecutive or male gender was a criterion. Intent-to-treat comparisons were employed in two studies [13–16]. Other biases that existed in the studies included non-uniform surgical projects.

### Incidence of HO at 1.5 months after surgery

**Fig. 4:**
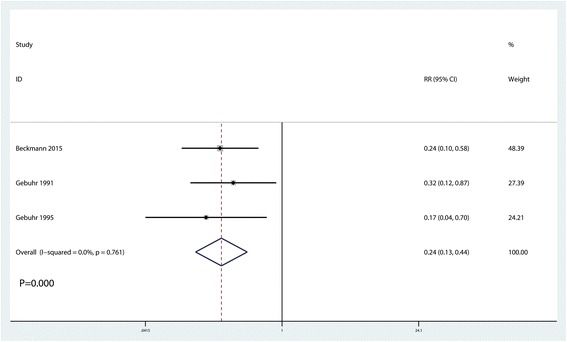
Forest plot comparing the occurrence of HO at 1.5 months after surgery between the two groups

The incidence of HO at 1.5 months after surgery was reported in three studies [13–16]. No heterogeneity between the included studies was found (*I*^2^ = 0.0%, *P* = 0.761). The pooled results indicated that use of naproxen was associated with reduced occurrence of HO at 1.5 months after surgery (RR = 0.247, 95% CI 0.13, 0.44, *P* = 0.000, Fig. 4).

### Incidence of HO at 3 months after surgery

**Fig. 5:**
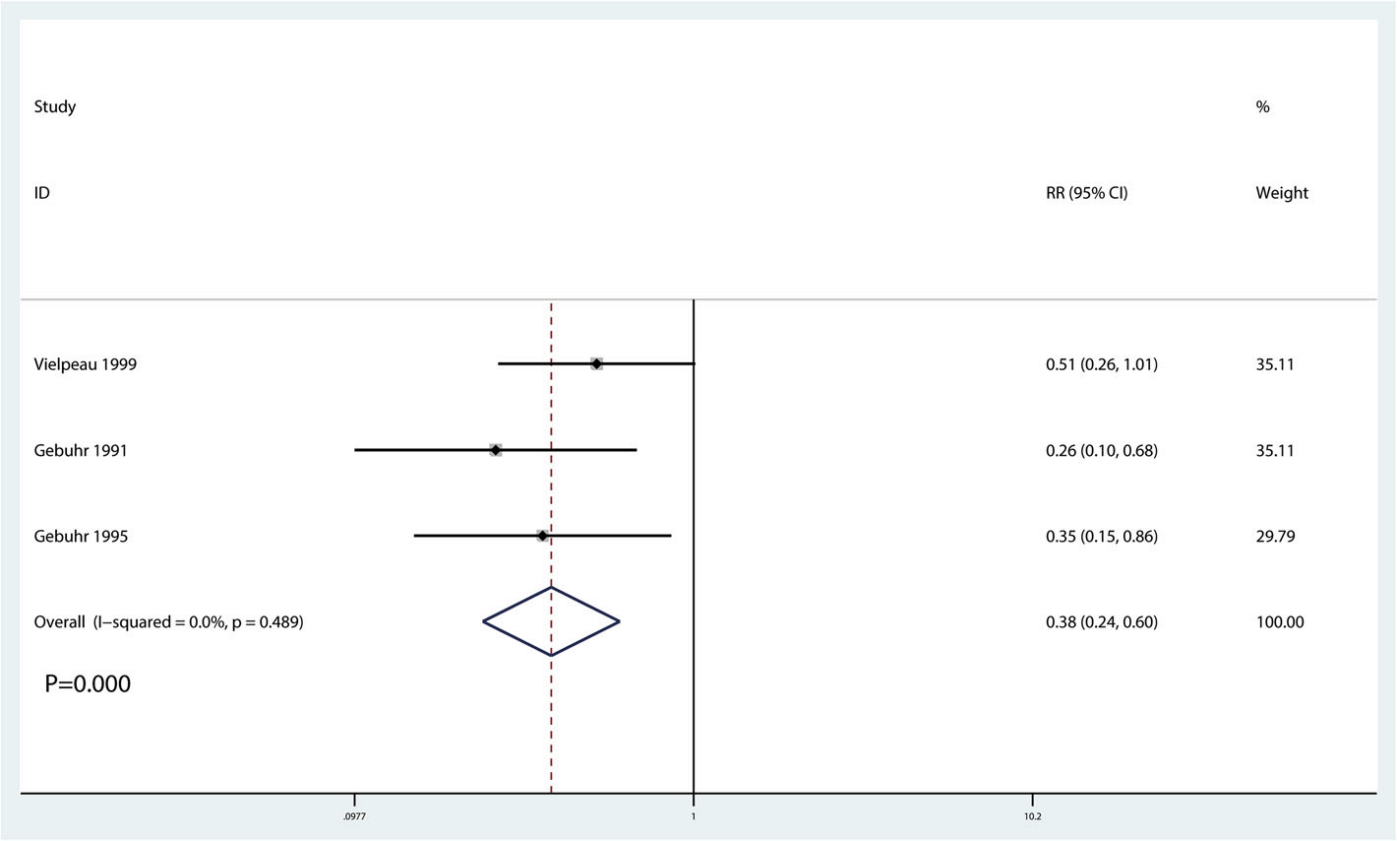
Forest plot comparing the occurrence of HO at 3 months after surgery between the two groups

Three studies reported the incidence of HO at 3 months after surgery [14–16]. Again, no heterogeneity was found (*I*^2^ = 4.0%, *P* = 0.353). The pooled results indicated naproxen administration to be associated with reduced HO occurrence at 3 months after surgery (RR = 0.35, 95% CI 0.21, 0.58, *P* = 0.000, Fig. 5).

### Incidence of HO at 6 months after surgery

**Fig. 6:**
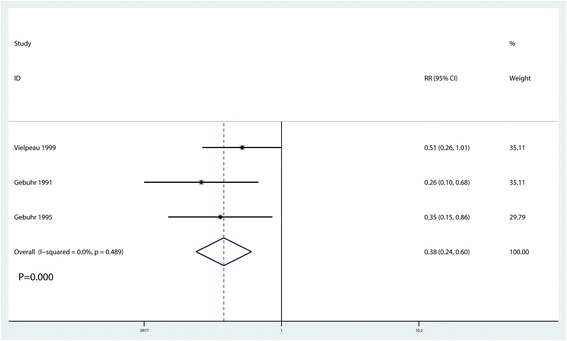
Forest plot comparing the occurrence of HO at 6 months after surgery between the two groups

Similarly, the incidence of HO at 6 months after surgery was reported in three studies [14–16], and no heterogeneity between the included studies was observed (*I*^2^ = 0.0%, *P* = 0.489). Based on the pooled results, administration of naproxen was associated with a reduction in the occurrence of HO at 6 months post-surgery (RR = 0.38, 95% CI 0.24, 0.60, *P* = 0.020, Fig. 6).

### Incidence of HO at 12 months after surgery

**Fig. 7:**
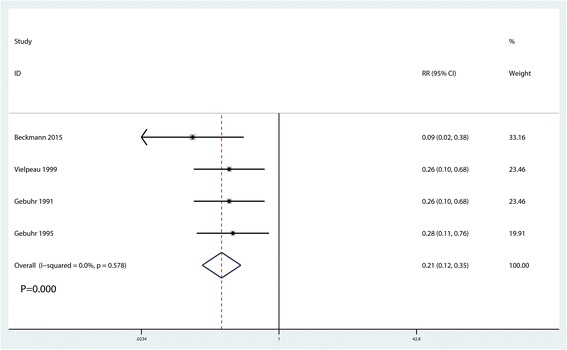
Forest plot comparing the occurrence of HO at 12 months after surgery between the two groups

All four studies reported the incidence of HO at 12 months after surgery [13–16]. There was no heterogeneity between the included studies (*I*^2^ = 0.0%, *P* = 0.578). According to the pooled results, naproxen administration was associated with decreases in the occurrence of HO at 12 months after surgery (RR = 0.21, 95% CI 0.11, 0.76, *P* = 0.000, Fig. 7).

### Complications

**Fig. 8:**
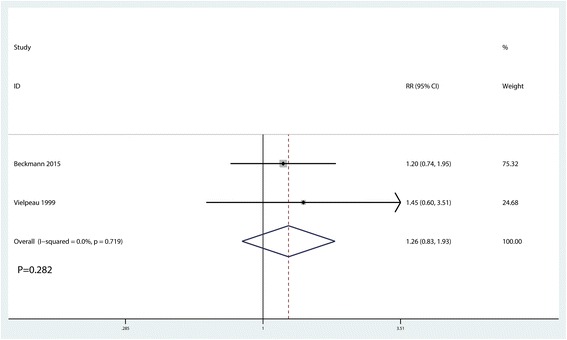
Forest plot comparing the occurrence of complications after surgery between the two groups

The incidence of complications after surgery was reported in two studies [13, 14], with no heterogeneity observed between the studies (*I*^2^ = 0.0%, *P* = 0.719). The pooled results indicated that use of naproxen had no impact on complications after hip surgery (RR = 1.26, 95% CI 0.83, 1.93, *P* = 0.282, Fig. 8).

## Discussion

This is the first meta-analysis that compares naproxen versus placebo for reducing the occurrence of HO after hip surgery. Based on the results of our meta-analysis, we found naproxen to be effective in reducing the occurrence of HO at 1.5-, 3-, 6-, and 12-month follow-ups. In addition, naproxen use did not increase the incidence of complications after hip surgery. The major strengths of our analysis include our systematic approach in identifying studies from PubMed, EMBASE, Web of Science, Cochrane Library, and Google databases and our rigorous data analysis.

We assessed the incidence of HO at 12 months after hip surgery, and the results showed that naproxen can reduce its occurrence. HO, which has many diverse causes and pathogenies, can be classified into the following three main types: genetic, neurogenic, and traumatic [17]. It is well known that higher grades of HO are associated with functional disabilities. Recently, clinically relevant HO has been defined as Brooker grade 3 or 4. The morbidity of definite HO following total hip arthroplasty (THA) ranges from 3 to 9%. We did not compare functional outcomes after surgery between the two groups because there was insufficient data for such an analysis.

In this meta-analysis, the two studies conducted by Gebuhr et al. taken together indicate that naproxen provided for either 4 weeks or 8 days is equally sufficient to decrease the incidence of HO [15, 16] Further studies are required to investigate shorter courses of treatment regimens following hip surgery. Although there is increasing evidence for the use of selective COX-2 inhibitors, which have been shown to produce fewer gastrointestinal side effects than NSAIDs, there are concerns due to the emergence of evidence of adverse cardiovascular complications, particularly in patients with cardiovascular disease or risk factors. For example, rofecoxib, a COX-2-inhibiting agent, has been withdrawn from the market. The Vioxx Gastrointestinal Outcomes Research (VIGOR) trial conducted by Bombardier indicated that rofecoxib can lower the incidence of clinically significant grades of HO, with lower rates of gastrointestinal toxicity but with higher incidence of myocardial infarction compared to naproxen [18]. Simultaneous prescription of mucoprotective agents with NSAIDs reduces gastrointestinal irritation, though this line of treatment should be reserved for patients who must avoid traditional NSAIDs (i.e., indomethacin and naproxen) due to a severe gastrointestinal disorder and for those who do not have significant cardiac risk factors.

In general, HO was assessed either via clinical outcomes using the Harris hip score and ROM or radiological outcomes using plain radiographs or Brooker classification [19]. By measuring the volume of heterotopic bone formation, other methods such as the 3-D computed tomography reconstructions used in Matta et al. may be more convincing for rating HO [20]. HO can typically be first diagnosed 6 to 12 weeks post-trauma [21]. It has also been reported that HO can be detected by ultrasound as early as 1 week following surgery [22]; thus, ultrasound may play a strong role not only in the early diagnosis of HO but also in adapting or commencing prophylactic therapy.

Serious complications of NSAID prophylaxis have been reported following hip surgery, including ototoxicity, renal failure, and hematochezia. However, there was no significant difference in the occurrence of complications between the naproxen and control groups (*P* > 0.05). Beckmann et al. [13] reported minor adverse reactions in 42% of patients taking naproxen and 35% of those taking placebo. Moreover, Vielpeau et al. [14] found that overall tolerance was rated as good by 87% of patients and 86% of physicians, with no difference between groups.

According to the evidence available, successful prophylaxis following hip surgery can be achieved using a course of naproxen ranging from 8 days to 6 weeks at a dose of 500 mg twice daily or 250 mg three times daily. Further studies are needed to evaluate whether naproxen has an advantage over other NSAIDs in the prevention of clinically significant HO.

Only four RCTs were included in this meta-analysis. Despite the great risk for publication bias, other published and unpublished data, administration of interventions, timing of applying naproxen, or methods of outcome assessment might result in significant differences. To guide its clinical application, more studies are needed to verify whether naproxen is more efficient than other NSAIDs for preventing clinical HO. Further efforts are needed to improve the clinical application of HO prophylaxis, for naproxen or other NSAIDs.

## Conclusions

In light of the positive effect of naproxen in reducing the occurrence of HO with no observed adverse impact on safety outcomes, naproxen may be used as an alternative to prevent HO after hip surgery. Further research is necessary to assess the impact of naproxen on decreasing the incidence of HO compared to other NSAIDs as well as to determine the optimal dose and treatment interval.

## Abbreviations

CI: Confidence interval
HO: Heterotopic ossification
NSAIDs: Nonsteroidal anti-inflammatory drugs
RCTs: Randomized controlled trials
ROM: Range of motion
RR: Risk ratio
VIGOR: Vioxx Gastrointestinal Outcomes Research
WMD: Weighted mean difference
